# The incidence of acute gastrointestinal illness in Sweden

**DOI:** 10.1177/1403494815576787

**Published:** 2015-07

**Authors:** Frida I Hansdotter, Måns Magnusson, Sharon Kühlmann-Berenzon, Anette Hulth, Kristian Sundström, Kjell-Olof Hedlund, Yvonne Andersson

**Affiliations:** 1 Department of Knowledge Development, Public Health Agency of Sweden, Sweden; 2 Department of Computer and Information Science, Linköping University, Sweden; 3 Department of Monitoring and Evaluation, Public Health Agency of Sweden, Sweden; 4 Institute for Food and Agricultural Economics, Lund University, Sweden; 5 Formerly at Swedish Institute for Communicable Disease Control, Sweden

**Keywords:** Estimating disease incidence, acute gastrointestinal illness, diarrhoea, public health, recall bias, syndromic surveillance, web query-based surveillance

## Abstract

*Aims:* The aim of this study was to estimate the self-reported domestic incidence of acute gastrointestinal illness in the Swedish population irrespective of route of transmission or type of pathogen causing the disease. Previous studies in Sweden have primarily focused on incidence of acute gastrointestinal illness related to consumption of contaminated food and drinking water. *Methods*: In May 2009, we sent a questionnaire to 4000 randomly selected persons aged 0–85 years, asking about the number of episodes of stomach disease during the last 12 months. To validate the data on symptoms, we compared the study results with anonymous queries submitted to a Swedish medical website. *Results*: The response rate was 64%. We estimated that a total number of 2744,778 acute gastrointestinal illness episodes (95% confidence intervals 2475,641–3013,915) occurred between 1 May 2008 and 30 April 2009. Comparing the number of reported episodes with web queries indicated that the low number of episodes during the first 6 months was an effect of seasonality rather than recall bias. Further, the result of the recall bias analysis suggested that the survey captured approximately 65% of the true number of episodes among the respondents. ***Conclusions*: The estimated number of Swedish acute gastrointestinal illness cases in this study is about five times higher than previous estimates. This study provides valuable information on the incidence of gastrointestinal symptoms in Sweden, irrespective of route of transmission, indicating a high burden of acute gastrointestinal illness, especially among children, and large societal costs, primarily due to production losses.**

## Introduction

Acute gastrointestinal illness (AGI) is a major public health problem worldwide and an important cause of mortality and morbidity. In children, diarrhoea is one of the leading causes of post neonatal deaths, estimated to have caused 800,000 deaths worldwide in children under 5 years of age in 2011 [1]. In high income countries, AGI associated mortality is low, but morbidity remains high. A review of estimates of the incidence and prevalence of AGI from 33 studies from high income countries has shown a range from 0.1 to 3.5 episodes per person year. However, comparisons of these rates are problematic due to variation in study design, mainly prospective cohort studies and retrospective cross sectional surveys, and should be made with caution [2].

In Sweden, AGI is generally a mild disease. Most affected persons need no health care but might stay home from work or school for a few days due to sickness or because of parents taking care of a sick child. As only some cases attend health care, and thus are diagnosed and captured in surveillance systems, special studies are needed to estimate the true incidence of AGI in the population. Estimating the disease burden and understanding the distribution of cases in the population is of importance for resource planning, the design of preventive strategies and for calculating costs.

Previous studies in Sweden have focused on incidence of AGI related to consumption of contaminated food and drinking water and have estimated the annual number of cases to 500,000 [3,4]. These estimates, however, only capture a part of the total number of AGI cases in the population. The aim of this study was to estimate the preventable domestic incidence of AGI in the Swedish population irrespective of route of transmission or type of pathogen causing the disease.

The study was part of a European collaborative burden-of-illness project developed within the European Union network of excellence on zoonoses Med-Vet-Net.

## Methods

### Study design and data collection

We conducted the study in May 2009 and included 4000 randomly selected persons aged 0–85 years from the population, who received a questionnaire by post. Our selection of study participants was made from Statens personadressregister (SPAR), a register including all persons who are registered as residents in Sweden. For study participants younger than 16 years of age, the questionnaire was addressed to a guardian. Study participants were free to choose between completing a paper questionnaire or a web-based questionnaire on the Internet using an individual access code. One reminder that included a new copy of the paper questionnaire and information about the study was sent out after 2 weeks.

The questionnaire collected information on the number of episodes of stomach disease during the last 12 months, that is, 1 May 2008 to 30 April 2009. For the last episode, we also asked for information on symptoms, number of sick days, health care consumption and out of pocket costs.

### Exclusion criteria

Episodes that started within 10 days of the participant having been abroad or within 6 months of the participant having had stomach, intestine or belly surgery or participants who reported recurrent problems with diarrhoea or any kind of chronic gastrointestinal disease such as Crohn’s disease were excluded from the analysis as these episodes were not considered preventable.

### Case definition

We defined an episode of AGI as either diarrhoea (three loose stools/24 hours) or at least three of the following symptoms: vomiting; stomach cramps; nausea; fever. We chose this case definition for the reason of comparability with previous unpublished studies in Sweden.

### Data analysis

To estimate the incidence rate of AGI per person year, we calculated the total number of episodes of AGI among the respondents by assuming that the same proportion of the episodes without information on symptoms met the case definition as among episodes with information on symptoms (last episode). We then divided the number of AGI episodes by the number of respondents in order to calculate the incidence rate of AGI episodes per person year.

We calibrated the total number of AGI episodes in the Swedish population with a generalised regression estimator where sex, age group and county were the auxiliary information of the weights [5]. This was done as response rates differed among subgroups and in order to ensure that the estimate reflected the population composition. The population data for this were those of 31 December 2008 and available from Statistics Sweden [6].

We fitted a negative binomial regression model to the number of AGI episodes per person with sex, age group and county of residence as explanatory variables. In this analysis, we did not use the weights obtained from the calibration.

### Data validation

In order to validate the data on symptoms, we compared the study results with data from a syndromic surveillance system developed and in routine use at the Public Health Agency of Sweden (former Swedish Institute for Communicable Disease Control). This system is based on anonymous queries submitted to a Swedish medical website (Vårdguiden.se) [7]. The data are collected in real time and reflect a point close to onset of any infectious disease based on the assumption that people search for information when falling ill, rather than when they have been ill for a while. Also, most persons who look for information on a medical website can be assumed to be ill and not search out of other reasons. From the system, it is possible to obtain time series of specified queries since 2005. The web queries cannot be used to estimate the number of episodes of gastroenteritis but can show seasonal variations. We extracted the number of queries on the Swedish equivalences to stomach disease, diarrhoea and vomit per month from 1 May 2007 to 30 April 2010.

### Analysis of recall bias

To determine if the incidence estimate was affected by recall bias, we fitted a model to the logarithm of the number of persons reporting at least one episode of stomach disease per month. We defined the model as a Gaussian generalised linear model with identity link and included as explanatory variables the month (starting on May 2008 (*t*=0) and ending on May 2009 (*t*=11)) and the monthly number of web queries, where the estimated effects were to be interpreted as proportions. We assumed the patterns of the web queries to follow the true incidence of stomach disease in the population, without any recall bias, thus describing the trend and seasonal effects of the true number of episodes. The hypothesis behind this is that if there is an additional increasing trend over time in the relationship between the reported number of episodes of stomach disease in this study and the number of web queries, this would be related to recall bias, where people remembered more recent episodes better. Since the number of degrees of freedom in the model was small, only strong recall bias effects would be possible to identify. We studied the residuals of the model with the Durbin–Watson (DW) test for auto correlated residuals. In the case of a significant recall bias, the percentage of episodes in the study was estimated by

(1) $$y=\frac{\sum_{t}x_{t}}{\sum_{t}\frac{1}{e^{\beta \left(t-12\right)}}*x_{t}}$$

where *y* is the estimated relative effect of the recall bias, *x_t_* is the number of episodes from the survey in month *t* and β is the coefficient for time in the regression model.

We analysed the data using the statistical software STATA 10 (StataCorp, USA); R [8] (version 2.13.0) including packages pscl [9,10], MASS [11] and survey [12,13] and Microsoft Office Excel 2007. The study was approved by the Regional Ethical Review Board in Stockholm, Sweden (reference 2009/525-31/2).

## Results

### Response rate

Of 4000 persons receiving the questionnaire, 2564 returned it, which implies a response rate of 64%. Men and younger age groups had lower response rates. Of 2564, 92% filled out the paper questionnaire and 8% filled out the web-based questionnaire. Five questionnaires were considered to be completed incorrectly and were excluded from further analysis. We also excluded another 65 questionnaires due to self-reported age differing more than ±1 years from the age obtained from SPAR since it was suspected that the wrong person had completed the questionnaire. In total we considered 2494 answers to be valid (Table I).

**Table I.** Number of population (31 December 2008) questionnaires sent out and respondents by age group and gender.

Age group (years)	Sex	Number (%) of population	Number (%) of questionnaires sent out	Number (%) of respondents
0–4	Female	260,995 (3)	91 (2)	67 (3)
	Male	275,987 (3)	114 (3)	75 (3)
5–14	Female	489,739 (5)	243 (6)	151 (6)
	Male	515,681 (6)	212 (5)	121 (5)
15–24	Female	595,206 (7)	287 (7)	155 (6)
	Male	626,537 (7)	265 (7)	124 (5)
25–39	Female	869,501 (10)	376 (9)	214 (9)
	Male	905,336 (10)	408 (10)	187 (7)
40–64	Female	1519,950 (17)	702 (18)	508 (20)
	Male	1552,334 (17)	638 (16)	394 (16)
65–85	Female	778,689 (9)	371 (9)	279 (11)
	Male	662,968 (7)	292 (7)	222 (9)
All female		4514,080 (50)	2070 (52)	1374 (55)
All male		4538,843 (50)	1929 (48)	1120 (45)
Total		9,052,923 (100)	4000 (100)	2494 (100)

### Incidence by age and gender

**Figure 1.**
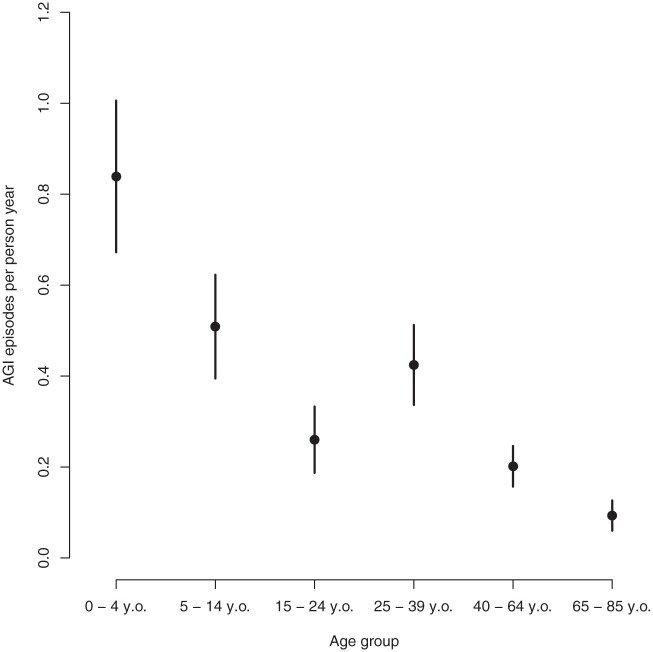
AGI incidence rate per person year per age group with 95% confidence intervals.

In total, 2453 respondents were included in the analysis and, of those, 562 reported in total 933 episodes of stomach disease during the last 12 months. Of the 562 episodes that included information on symptoms (last episode), 451 met the AGI case definition, i.e. 80%. Assuming the same proportion of AGI episodes among those episodes without information on symptoms, the incidence rate of AGI episodes per person year was 0.31 (95% confidence intervals (CI) 0.28–0.34). Sex did not seem to have an effect on incidence rate (*p*-value=0.21): the number of AGI episodes per person year among males was 0.25 and among females was 0.33. We did not identify any statistically significant differences in disease between counties in the regression model when also including sex and age (*p*-value=0.11). The incidence rate was, however, affected by age with 0.84 (95% CI 0.67–1.00) AGI episodes per person year in the age group 0–4 and 0.09 (95% CI 0.06–0.13) in persons older than 65 years (Figure 1). We did not find any significant interaction between age and sex except in the age group 25–39, where females had a higher incidence rate (0.55) than males (0.30) (*p*-value=0.02).The calibrated estimate of total number of AGI episodes in the Swedish population was 2744,778 (95% CI 2475,641–3013,915) episodes between 1 May 2008 and 30 April 2009 (Table II).

**Table II.** Estimated total number of AGI episodes between 1 May 2008 and 30 April 2009 in Sweden, by age group.

	Point estimate	95% confidence interval	
		Lower	Upper
Total	2744,778	2475,641	3013,915
0–4 years	450,580	361,053	540,106
5–14 years	509,340	395,128	623,552
15–24 years	309,003	222,211	395,795
25–39 years	734,393	581,838	886,948
40–64 years	609,601	474,518	744,684
65–85 years	131,861	84,807	178,915

### Description of AGI cases

#### Symptoms

Of the 451 cases who described their last AGI episode in detail, 242 (54%) reported diarrhoea and vomiting, 134 (30%) reported diarrhoea and no vomiting and 73 (16%) reported vomiting and no diarrhoea. Two cases reported light symptoms with a combination of nausea, fever and stomach cramps. Of the episodes with diarrhoea, 3% (10/376) indicated bloody diarrhoea.

#### Health care consumption

In 12% of the AGI cases, the individuals sought counsel from a nurse about their symptoms through a public telephone service (Sjukvårdsrådgivningen) open 24 hours per day; 12% sought information on the Internet; and 6% used both means. Moreover, 9% (39 out of 449 who provided a reply to this question) sought health care, out of which 18% (7/39) were hospitalised for a median number of 2 days (range 1–14 days). Three of the seven hospitalised cases were in the age group of 0–4 years. Of all cases that sought health care, 41% (15/37) reported that they had left faeces sample during their health care visit. Of the cases that indicated bloody diarrhoea, 60% sought health care. Median duration of illness for all cases was 3 days (range 1–40 days).

#### Sick leave

Of the 451 cases who described their last AGI episode in detail, 74% reported staying home from work or school due to illness during a median number of 3 days (range 1–30 days). In Sweden, parents can obtain temporary parental benefit when staying home from work to look after a sick child under the age of 12. Of the parents of cases under the age of 12, 81% stayed home from work to take care of the sick child for a median number of 3 days (range 1–15 days).

#### Costs

Only 125 cases provided details about out of pocket costs related to the illness (loss of income excluded). The median medical costs were €8 (range €0–207) and the median of other costs was €7 (range €0–310). The main type of transportation used to go to the general practitioner surgery or hospital was by car, which was used in 73% of the trips with a median distance of 36 km (range 2–200 km) both ways included.

### Recall bias

**Figure 2.**
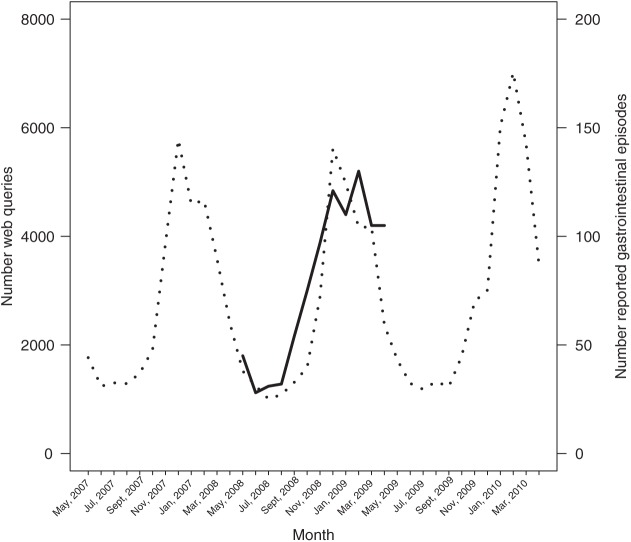
Number of reported gastrointestinal episodes in thousands per month between 1 May 2008 and 30 April 2009 (solid line, right y-axis) and number of web queries for stomach disease, diarrhoea or vomit (dotted line, left y-axis) between 1 May 2007 and 30 April 2010.

A vast majority of all reported episodes of stomach disease were reported to have occurred during the last 6 months of the recall period, i.e. between 1 November 2008 and 30 April 2009, which could be an effect of recall bias. From the syndromic surveillance system based on web queries, it has previously been noted that queries on vomit have a strong seasonality, while queries on diarrhoea are evenly distributed over time [14]. In the study, we used web queries on (the Swedish equivalents to) stomach disease, diarrhoea and vomit. The total number of queries per month on these three symptoms varied between 1005 in July 2008 and 7017 in February 2010, with a median of 2140 queries per month. When comparing the results of the reported episodes of stomach disease (gastrointestinal symptoms) with the included web queries, we found that the reported episodes followed the same pattern as the web queries (Figure 2). This finding can explain why the vast majority of the episodes were reported to have occurred between November 2008 and April 2009 and were not necessarily due to an effect of recall bias. However, in exploring possible recall bias, we found that the number of reported episodes of stomach disease was significantly associated with both the web queries (${\hat{b}}_{web}$=0.00016, *p*=0.03) and time (${\hat{b}}_{month}$=0.095; *p*=0.01).Thus, after taking into account the effect of the pattern of the web queries, it still remained that the logarithm of the number of reported episodes increased approximately by 10% per month, which we interpret as a result of better recall closer to the date of the survey. We did not find any statistically significant autocorrelation in the model (DW statistic=1.38, *p*=0.082).

**Figure 3.**
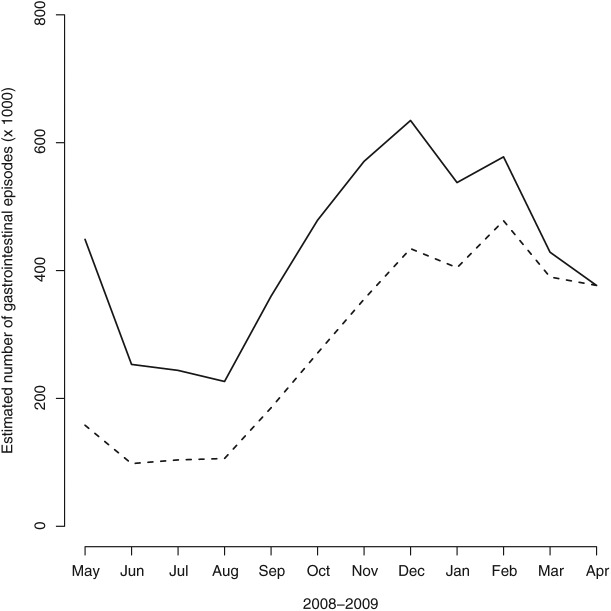
Estimated number of gastrointestinal episodes (dotted line) from model and estimated number of gastroenteritis episodes corrected for recall bias (solid line) both in thousands and per month between 1 May 2008 and 30 April 2009.

Based on the results from the model, we concluded that only 65% of the episodes of gastroenteritis during the survey period were identified in this study. Figure 3 shows the number of episodes of stomach disease reported per month as well as a time series corrected for recall bias based on this model. It can be noted that the seasonal variation is still strong in the corrected data.

## Discussion

In this survey we estimated the domestic incidence rate of AGI in the Swedish population to be 0.31 (95% CI 0.28–0.34) per person year, irrespective of route of transmission or type of pathogen causing the disease. We estimated that a total number of 2744,778 episodes (95% CI 2475,641–3013,915) occurred during the study period (1 May 2008 to 30 April 2009), which is about five times higher than previous estimates made in Sweden focusing on incidence of AGI related to only consumption of contaminated food including drinking water [3,4]. We did not find any differences in disease incidence between women and men. This is in line with results from Denmark [15] but differs from other studies, where higher incidence has been reported in women compared to men [16–18]. We found the highest incidence in the youngest age group and decreasing by age, which has also been reported from other studies [15,16,19]. Only 9% sought health care but 74% reported staying home from work or school due to illness for a median number of 3 days. A further 81% of parents to cases under the age of 12 stayed home from work to take care of the sick child for a median number of 3 days. This indicates large societal costs due to AGI in the population related to production losses.

The seasonality of self-reported AGI might be explained by a high number of norovirus cases during the winter and early spring [14].The number of Salmonella cases infected in Sweden is low compared with most other countries but increases during the summer and early autumn like most other bacterial AGIs, such as indigenous Campylobacter infections [20].

It has been shown that the recall period used in retrospective studies heavily influences the estimates of AGI, resulting in higher rates of AGI when using a 7-day recall period compared with a 1 month recall period [15,21,22]. One possible error in retrospective studies is telescoping, which might result in higher incidence estimates as respondents remember episodes to have occurred more recently than when they actually did [23]. Further, if the recall period is too long, symptoms are likely to be under-reported as study participants might forget episodes that have occurred. As differences in study methodologies heavily influence the incidence estimates, comparisons between studies should be made carefully. Keeping this in mind, the incidence results from our study are within the range of what has been found in other high-income countries [2].

There are a number of limitations to this study. The case definition used is generous, which might lead to an overestimation of AGI occurrence. However, only 16% of the AGI cases reported vomiting and no diarrhoea and only two cases reported light symptoms with a combination of nausea, fever and stomach cramps, according to the last episode symptoms. Using different case definitions complicates comparison between studies, which is why future studies in Sweden would benefit from using the common symptom-based case definition for gastroenteritis recommended by Majowicz et al. [24]. Also, respondents who recently had gastroenteritis may have been more prone to answer (and to recall previous episodes of AGI) compared to respondents without symptoms, which might have led to an overestimation of the incidence. On the other hand, the long recall period of 12 months likely led to an underestimation of the incidence estimate due to cases forgetting their episodes.

In the presented study, we used web queries to validate results obtained from a survey. More precisely, we used the web queries to correct for any potential recall bias regarding the respondents’ stated symptoms. However, in the survey only 12% of those who experienced any AGI symptoms stated that they had used the Internet to look for health information. This means that the picture we get from the web queries is only partial. Also, we cannot be sure that the persons looking for information on the Internet actually are experiencing the symptoms in question. Although data on web queries have their limitations, we believe an analysis of web queries can contribute with important information related to the seasonality of self-reported burden of AGI in a society. A survey, on the other hand, enables us to collect detailed information on each case, such as symptoms, health care seeking behaviour and sick leave.

A 12-month recall period was used in this study. As the recall period was long, we believe the AGI was under-reported. However, in this study the recall bias was possible to explore in an innovative way by comparing our results to data on web queries on symptoms during the study period. When comparing the number of reported episodes with data that can be assumed to reflect a point close to onset, it was clear that the low number of episodes during the first 6 months was mainly an effect of seasonality rather than recall bias. Further, the result of the recall bias analysis suggests that the survey captured approximately only 65% of the true number of episodes. To our knowledge, this is the first time that non-specific surveillance data, in this case web queries, were used to validate self-reported previous episodes of gastrointestinal illness. The results are promising, as the web query data are supposed to give a retrospective picture of the disease patterns in society without interference of recall bias and the methods should be further explored.

This study provides valuable information on the incidence of gastrointestinal symptoms in Sweden, irrespective of route of transmission, indicating a high burden of AGI, especially among children, and large societal costs primarily due to production losses. Detailed information collected on health care seeking behaviour, costs, number of sick days and staying home from work will be useful for future cost calculations related to AGI and to design prevention activities.
